# Genotoxicity and antigenotoxicity study of aqueous and hydro-methanol extracts of *Spondias mombin* L., *Nymphaea lotus* L. and *Luffa cylindrical* L. using animal bioassays

**DOI:** 10.1515/intox-2015-0028

**Published:** 2015-12

**Authors:** Ifeoluwa Temitayo Oyeyemi, Olaide Maruf Yekeen, Paul Olayinka Odusina, Taiwo Mary Ologun, Orezimena Michelle Ogbaide, Olayinka Israel Olaleye, Adekunle A. Bakare

**Affiliations:** Cell Biology and Genetics Unit, Department of Zoology, Faculty of Science, University of Ibadan, Ibadan, Nigeria

**Keywords:** cytogenotoxicity, micronucleus, medicinal plants, acute toxicity, sperm morphology

## Abstract

*Spondias mombin* (Linn), *Nymphaea lotus* (Linn) and *Luffa cylindrica* (Linn) (syn *Luffa aegyptiaca* Mill) are plants traditionally used as food ingredients and in the management of diseases, including cancer, in Nigeria. Despite the therapeutic potentials attributed to these plants, reports on their genotoxicity are scanty. In this study, the genotoxicity of the aqueous and hydro-methanol extract of these plants was evaluated using mouse bone marrow micronucleus and sperm morphology assays. Antigenotoxicity was assessed by the bone marrow micronucleus test. The highest attainable dose of 5 000 mg/kg according to OECD guidelines was first used to assess acute toxicity of the aqueous and hydro-methanol extracts in Swiss albino mice. For each extract, there were five groups of mice (n=4/group) treated with different concentrations of the extract as against the negative and positive control group for the genotoxicity study. In the antigenotoxicity study, five groups of mice were exposed to five different concentrations of the extracts along with 60 mg/kg of methyl methane sulfonate (MMS), which was used to induce genotoxicity. The mice were administered 0.2 mL of extract per day for 10 days in the genotoxicity and antigenotoxicity groups. Administration of each of the extracts at the concentration of 5 000 mg/kg did not induce acute toxicity in mice. At the concentrations tested, all the extracts, except aqueous S. *mombin*, increased micronucleated polychromatic erythrocytes. The aqueous and hydro-methanol extracts of *N. lotus* increased the frequency of aberrant sperm cells. All the extracts were also able to ameliorate MMS induced genotoxicity in bone marrow cells of the exposed mice. The results showed the potential of the extracts to induce somatic and germ cell mutation in male mice. The extracts also ameliorated the genotoxic effect of MMS.

## Introduction

The medicinal use of plants has always been part of human practice and it may be as old as humankind itself. It has been estimated that between 60–90% of the populations of developing countries use traditional and botanical medicines almost exclusively and consider them to be a normal part of primary healthcare (WHO, 2002). The interest in the use of medicinal plants is based on the assertion that they contain natural substances that can promote health and alleviate illness. They are considered to be safe, are relatively less expensive, globally competitive and patients tolerate them well (Partap *et al*., 2012a). It is estimated that about 25% of all modern medicines are directly or indirectly derived from plants (Craig *et al*., 1997).

However, despite the profound therapeutic advantages possessed by some of the medicinal plants, some constituents of medicinal plants have been shown to be potentially toxic, mutagenic, carcinogenic and teratogenic (Oyedare *et al*., 2009; Schmeiser *et al*., 2009; Lather *et al*., 2011; van den Berg *et al*., 2011; Cuyacot *et al*., 2014; Ferreira-Machado *et al*., 2014). Severe side effects such as cardiovascular toxicities, neurotoxicity, diarrhea, cramps, dermatitis, allergic reactions have been reported (Lather *et al*., 2011). Moreover, most of the traditional medicinal plants have not been subjected to exhaustive toxicological tests as required for modern pharmaceutical compounds.

The evaluation of toxic, cytotoxic and genotoxic damage caused by plant compounds is of fundamental importance in minimizing the possible risks of these agents, especially when they are part of long-term treatment (Rodeiro *et al*., 2006). Plants exhibiting clear mutagenic properties should be considered potentially unsafe and thus they require further testing before their continued use can be recommended. In contrast, plants with obvious antimutagenic potential can be considered interesting for therapeutic use and merit further investigation into their pharmacological properties. Natural antimutagenic substances are particularly interesting as they are assumed to lower the cancer risk from everyday exposure to environmental and pharmaceutical mutagens (Verschaeve & Staden, 2008).

*Spondias mombin* (Linn), *Nymphaea lotus* (Linn) and *Luffa cylindrica* (Linn) (syn *Luffa aegyptiaca* Mill) are commonly used medicinal plants in the traditional management of cancer in Nigeria. *S. mombin* has been reported to show biological activities such as anxiolytic (Ayoka *et al*., 2005), anti-epileptic and anti-psychotic (Ayoka *et al*., 2006), anticonceptive (Uchendu & Isek, 2008), hepatoprotective (Hamenoo, 2010), cardioprotective (Akinmoladun *et al*., 2010), anti-inflammatory (Nworu *et al*., 2011), leish-manicidal (Accioly *et al*., 2012), learning and retention improving (Asuquo *et al*., 2013a), lactogenic (Akouédégni *et al*., 2013), hematinic (Asuquo *et al*., 2013b), antifertility (Asuquo *et al*., 2013c) and oxytocic (Nworu *et al*., 2007; Pakoussi *et al*., 2013) effects and was also found to prevent post-partum hemorrhage (Pakoussi *et al*., 2013). It was further reported to have blood lipid-lowering (Igwe *et al*., 2008), anti-free radical and anti-aging activities and to reduce glutathione synthesis (Pauly & Fleury, 2002). It was found to exhibit beta lactamase inhibitory activity (Coates *et al*., 1994) and a-amylase inhibitory activity (Fred-Jaiyesimi *et al*., 2009).

*Nymphaea lotus* has been reported to possess antibacterial (Akinjogunla *et al*., 2010), antidiabetic (Chaurasia *et al*., 2011) and antioxidant (Afolayan *et al*., 2013) effects. *L. cylindrica* was shown to possess anti-inflammatory (Muthumani *et al*., 2010; Khan *et al*., 2013; Kanwal *et al*., 2013), immunostimulatory (Mao *et al*., 2004), oxytocic (Kamatenesi-Mugisha *et al*., 2007), bronchodilatory (Muthumani *et al*., 2010), antidiabetic (Hazra *et al*., 2011), antibacterial (Muthumani *et al*., 2010, Ahmad & Khan, 2013), antifungal and phytotoxic (Ahmad & Khan, 2013), anti-asthma, anti-tussive and expectorant (Partap *et al*., 2012b), hepatoprotective (Balakrishnan & Huria, 2011; Pal & Manoj, 2011), antioxidant (Du *et al*., 2006; Prakash *et al*., 2010; Balakrishnan & Sharma, 2013), analgesic, antipyretic (Balakrishnan and Sharma, 2013) and antiemetic (Khan *et al*., 2013; Kanwal *et al*., 2013) activities.

Despite the therapeutic potentials attributed to these plants, reports on their genotoxicity are scanty. We reported the cytogenotoxic effects of aqueous extracts of these plants in *Allium cepa* (Oyeyemi & Bakare, 2013). In the present study, two eukaryotic mutagenicity assays, namely the micronucleus (MN) test in mouse bone marrow cells and the mouse sperm morphology assay, were used to evaluate the genotoxic and antigenotoxic potential of the aqueous and hydro-methanol extracts of *S. mombin, N. lotus* and *L. cylindrica.* These assays were chosen because they are *in vivo* assays in animal models to evaluate the genotoxic and antigenotoxic effects of selected plant extracts in somatic and germ cells. They are standard genotoxicity bioassays that reflect the delicate balance between pathways for activation and inactivation of chemicals in human beings and are well-known techniques to quantify genomic instability induced by chemical compounds.

## Materials and methods

### Collection and identification of plants

The leaves of *S. mombin*, known as hog plum in English and commonly called ‘Iyeye’ in Yoruba language of Nigeria, whole plants of *N. lotus* known as water lilly in English and commonly called ‘Osibata’ in Yoruba language of Nigeria, and the fruits of *L. cylindrica* known as Loofah in English and called ‘Kankan ayaba’ in Yoruba language of Nigeria, were collected at different locations within the premises of the University of Ibadan, Nigeria. They were taken to the University of Ibadan Herbarium for identification and authentication and voucher specimens (*S. mombin* UIH-22350, *N. lotus* UIH-22349, *L. cylindrica* UIH-22348) were deposited. The names of the plants were also checked with www.theplantlist.org *(S. mombin* 71480-1, *N. lotus* 605604-1, *L. cylindrica* syn *L. aegyptiaca* 293060-1). The plant parts were washed with tap water, dried in shade, ground and stored in the dark.

### Extraction

Aqueous and hydro-methanol crude extracts were prepared for each of the three plants. Aqueous extracts were prepared by boiling 100 g of dried grounded plant material in 2 L of tap water. The resultant decoctions were filtered with Whatman^®^ No. 1 (Maidstone, UK; 11 μm) filter paper. Hydro-methanol extraction was carried out by simple maceration process. Plant materials were soaked in 80% methanol for 72 hours with constant manual shaking. The resultant mixture was filtered with Whatman^®^ No. 1 (Maidstone, UK; 11 μm) filter paper. In both aqueous and hydro-methanol extracts, solvents were evaporated at 40 °C and pressure reduced using a rotary evaporator and then kept at 4 °C until use. The aqueous extracts of *S. mombin, N. lotus* and *L. cylindrica* were designated ASM, ANL and ALC, respectively, while the hydro-methanol extracts were designated as MSM, MNL and MLC, respectively. The reconstituted extracts were again filter sterilized using 0.22 μm sterile membrane filter (MILLEX’GP) before given to the animals.

### Biological material

Young male Swiss albino mice *(Mus musculus*, 6- and 10–11-weeks old) which had been inbred for several generations, were obtained from the animal breeding unit of the Department of Zoology, University of Ibadan, Nigeria and were maintained as an inbred colony. They were kept in a pathogen free, well ventilated section of the animal house at the Department of Zoology, University of Ibadan. They were maintained in the same room throughout the period of this study. Food (Ladokun pelleted feed^®^) and drinking water were supplied *ad libitum.* Mice, 8 weeks of age, were used for the acute toxicity and MN tests, while 12–14-week-old mice were used for the sperm morphology assay. All experiments were carried out in accordance with guidelines for the care and use of laboratory animals by the National Institute of Health, and approved by the University of Ibadan Animal Care and Use Research Ethics Committee (UI-ACUREC/App/2015/019).

### Acute toxicity test

For each extract there were two groups of mice (n=5). One group served as unexposed control and the other group received 5 000 mg/kg of the extract. The highest attainable dose (5 000 mg/kg) according to OECD guidelines for acute toxicity tests was used, because lower doses of these extracts have been reported to be non-toxic in rodents (Ayoka *et al*., 2005; Uchendu & Isek, 2008; Hamenoo, 2010; Hazra *et al*., 2011). The animals (n=5) were fasted overnight and then administered a single dose of 5000 mg/kg of each extract orally. They were observed for behavioral changes, signs of toxicity and mortality for the first 4 hours and thereafter daily over 14 days (Hwang *et al*., 2013).

### MN test

The MN test was used to assess the somatic genotoxicity and antigenotoxicity of each of the extracts. For the genotoxicity assessment, four groups of mice (four mice per group, weight range of20–25 g) per extract were used. Each group for each of the aqueous extracts corresponded to concentrations of200 mg/kg, 400 mg/kg, 800 mg/kg and 1600 mg/kg of each plant sample as against the negative (distilled water) and positive [60 mg/kg Methyl methane sulfonate (Sigma Aldrich USA 129925), intraperitoneally] controls. For the hydro-methanol extracts, each group corresponded to concentrations of 50 mg/kg, 100 mg/kg, 200 mg/kg and 400 mg/kg of each plant sample as against the negative (distilled water), vehicle solvent [20% dimethyl sulfoxide (DMSO) for MSM and 1% DMSO for MNL and MLC] and positive (60 mg/kg MMS, intraperitoneally) controls. Each mouse per group was orally (p.o.) administered 0.2 mL of plant extract per day for 10 consecutive days. Bone marrow preparation for MN assessment was carried out according to Schmidt (1976) and Alabi and Bakare (2011). For the antigenotoxicity study, the mice were treated as in the genotoxicity group but in the extract groups each mouse received a single dose of MMS (60 mg/kg) on the last day of extract treatment. The animals were sacrificed by cervical dislocation. The femurs were removed and bone marrow flushed from the bones with Fetal Bovine Serum (PAA Laboratories GmbH, Austria). Cells were centrifuged at 2 000 rpm for 5 minutes and slides were stained with May-Grunwald and Giemsa stains. At least 1 000 cells per mouse were scored at x1 000 for MN in polychromatic erythrocytes (MNPCE) and normochromatic erythrocytes (MNNCE).

### Sperm morphology assay

In this assay, the same number of groups, types of samples, treatment and controls as in the genotoxicity study with micronucleus assay were used. Five mice were treated in each concentration in a 5-week exposure period. At 5 weeks from the first day of exposure, the mice were sacrificed by cervical dislocation and their caudal epididymes were surgically removed. Sperm smears were prepared from the epididymes as previously described (Wyrobek *et al*., 1983; Alabi & Bakare, 2011). For each mouse, 1000 sperm cells were assessed for morphological abnormalities according to standard (Wyrobek & Bruce, 1975; Wyrobek *et al*., 1983; Bakare *et al*., 2005; Alabi & Bakare, 2011).

### Statistical analysis

All results were expressed as mean±SE or % frequency and were analyzed by SPSS for Windows, version 18.0 (SPSS Inc., Chicago, IL). PCE, NCE, and MNPCE percentages were calculated and the ratio of PCE to NCE was also recorded. Statistical comparisons were performed by one-way analysis of variance (ANOVA) followed by Duncan's multiple range test. The results were considered significant if the p-values were 0.05 or less.

## Results

### Acute toxicity

Oral administration of the extracts tested did not cause death in the mice at the concentration (5000 mg/kg) given over fourteen days. There was no change in behavioral activities.

### MN assay

**Figure 1:**
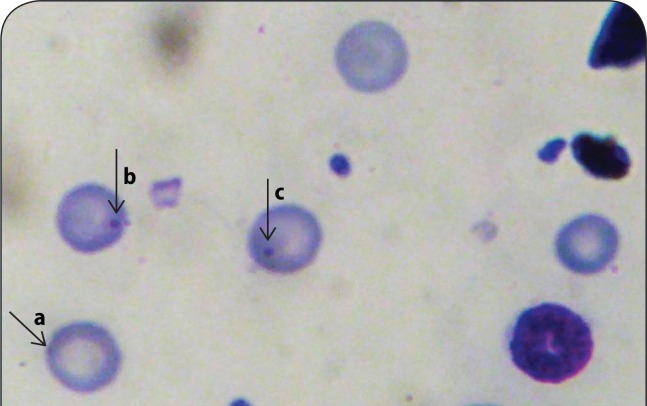
Micronuclei (MN) induced in mice exposed to aqueous and hydro-methanol extracts of *S. mombin, N. lotus* and *L. Cylindrica.* (a) Normal polychromatic erythrocyte (PCE) (b & c). Micronucleated polychromatic erythocyte (MNPCE); Magnification 1000x.

Table 1 shows the frequencies of MNPCE and % PCE (Figure 1) observed in bone marrow of mice exposed to the aqueous plant extracts in the genotoxicity and antigenotoxicity studies. In the genotoxicity study, a significant increase in MNPCE was observed in the aqueous extract groups except at the 400 and 1 600 mg/kg concentrations of ASM. Likewise in the antigenotoxicity study, a significant increase in MNPCE compared to negative control was observed except at the 1600 mg/kg of ASM. There was however a reduction in MNPCE compared to the MMS group; (55.6–83.7% reduction in ASM group, 42.0–74.1% reduction in ANL group and 23.8–77.4% reduction in ALC group).

**Table 1:** Frequencies (Mean±SE) of micronucleated erythrocytes (% MNEs) and mitotic activity (% PCE) in bone marrow of mice exposed to aqueous extracts of *Spondias mombin* (ASM), *Nymphaea lotus* (ANL) and *Lufffa cylindrica* (ALC) alone and with methyl methane sulfonate

Conc. (mg/kg)	MNPCE	ASM % Amelioration	% PCE	MNPCE	ANL % Amelioration	% PCE	MNPCE	ALC % Amelioration	% PCE
DDW	4.8±0.8		61.4±1.8	4.8±0.8		61.4±1.8	4.8±0.8		61.4±1.8
200	10.0±0.4^††^		54.2±1.2^†^	8.3±2.9^††^		68.5±0.7^†^	12.8±1.6^††^		49.2±5.4^†^
400	2.5±1.0		54.6±0.8^†^	11.0±2.3^††^		59.3±4.2	9.3±1.5^††^		59.5±0.8^†^
800	5.5±1.2^†^		51.8±0.2^††^	8.5±1.2^††^		59.3±0.3	18.7±5.5^††^		54.5±3.1
1600	4.0±1.5		52.0±1.8^††^	13.3±0.3^††^		55.7±1.4	29.0±3.0^††^		58.1 ±0.9
MMS	28.0±3.1^††^		46.3±0.6^††^	28.0±3.1^††^**		46.3±0.6	28.0±3.1^††^		46.3±0.6^††^
200+MMS	12.0±0.4^††^**	55.6	57.7±1.5**	16.3±2.8^††^**	42.0	54.0±0.5^††^*	20.5±1.3^††^*	23.8	60.4±0.2**
400+MMS	6.5±1.2^††^**	75.0	59.4±1.7**	12.0±3.2^††^**	57.1	54.9±1.7^†^**	7.3±1.6^†^**	74.1	55.9±1.2*
800+MMS	7.0±1.8^††^**	73.1	57.4±0.5**	7.3±1.1^††^	74.1	60.7±0.3**	6.0±1.7^†^**	77.4	59.1±1.6**
1600+MMS	4.3±1.0**	83.7	40.0±3.4^†^*	10.7±2.7^††^**	61.9	50.1±3.2^††^*	17.5±0.5^††^**	32.7	50.7±2.3^†^

All the groups were compared with the negative control. The antigenotoxic groups were also compared with MMS. Significant difference from distilled water group

† -(*p*<0.05)

†† -(*p*<0.01); Significant difference from MMS

* *-(*p*<0.05)

** -(*p*<0.01); DDW-distilled water; MMS-methyl methane sulfonate (60 mg/kg); MNPCE-micronucleated polychromatic erythrocyte; PCE-polychromatic erythrocyte.

The % PCE compared to the negative control was significantly lower in the ASM treated groups, while it was insignificantly lower in the ASM+MMS treated groups, except at the 1600 mg/kg dose (Table 1). In the ANL treated group, % PCE was significantly higher in the 200 mg/kg group compared to the negative control, while an insignificantly lower concentration dependent % PCE was observed at the other concentrations. In the ANL+MMS, the % PCE was significantly lower except at 800 mg/kg. In the ALC and ALC+MMS group, the % PCE was insignificantly lower in all the groups except the 200 and 400 mg/kg groups.

Table 2 shows the results of MNPCE and % PCE observed in bone marrow of mice exposed to the hydromethanol extracts. Compared to the vehicle control, significant increase in the frequency of MNPCE was observed at 100, 200 and 400 mg/kg concentrations of MSM, 100 mg/kg of MNL and 100 and 200 mg/kg of MLC. However, compared with the negative control, there was a significant increase in frequency of MNPCE at all the concentrations tested of the three plants. In the antigenotoxicity study, compared to the negative and vehicle controls, there was a significant increase in MNPCE observed at the concentrations of MSM tested, but the MNPCE frequency was also significantly lower compared to the positive control for the hydro-methanol plant extracts tested.

**Table 2:** Frequencies of micronucleated erythrocytes (% MNEs) and mitotic activity (% PCE) in bone marrow of mice exposed to hydro-meth-anol extracts of *Spondias mombin* (MSM), *Nymphaea lotus* (MNL) and *Lufffa cylindrica* (MLC) alone and with methyl methane sulfonate MMS (Mean±SE).

Conc. (mg/kg)	MNPCE	MSM % Amelioration	% PCE	MNPCE	MNL % Amelioration	% PCE	MNPCE	MLC % Amelioration	% PCE
DDW	4.8±0.8		61.4±1.8	4.8±0.8		61.4±1.8	4.8±0.8		61.4±1.8
DMSO	8.8±3.4^††^		55.3±1.5^††^	8.75±1.0^††^		60.2±4.8^††^	8.75±1.0^††^		60.2±4.8
50	7.0±1.8^††^		57.1±1.6^††^^‡^	9.5±1.2^††^		57.1±0.8^††^	13.67±3.0^††^^‡‡^		64.5±3.3
100	13.7±3.8^††^^‡‡^		54.9±0.6^††^	13.8±0.9^††^^‡^		58.0±1.6^††^	17.25±2.4^††^^‡‡^		55.7±0.2
200	19.0±1.0^††^^‡‡^		54.8±0.1^††^	7.5±1.8^††^		66.0±0.7^††^	7.25±1.4^††^		55.1±0.8
400	16.0±3.5^††^^‡‡^		53.0±0.9^††^	11.5±2.5^††^		66.7±0.8^††^	8.0±1.0^††^		61.1±0.9
MMS	28.0±3.1^††^^‡‡^		46.3±0.6^††^^‡‡^	28.0±3.1^†††^^‡^		46.3±0.6^††^	28.0±3.1^††^^‡‡^		46.3±0.6
50+MMS	9.0±0.7^††^^‡^**	57.1	49.4±0.5^††^	12.8±1.1^††^**	39.3	55.2±0.7^††^	6±0.0^††^**	78.8	55.7±0.2
100+MMS	9.0±0.7^††^^‡^**	57.1	51.1±3.5^††^**	8.5±1.2^††^**	59.5	55.5±1.9^†^	14.25±.1.5^†^^‡‡^**	44.1	56.2±0.0
200+MMS	11.3±0.6^††^^‡‡^**	46.4	51.6±0.9^††^*	6.0±1.2^††^**	71.4	69.5±1.2^††^	6.5±1.5^††^**	75.0	52.8±3.5
400+MMS	9.0±3.5^††^^‡^**	56.0	59.1±1.1^†^^‡^**	6.5±1.5^††^**	69.1	54.1±1.6	5.5±1.5**	76.2	52.1±6.1

All the groups were compared with the negative and vehicle control. The antigenotoxic groups were also compared with MMS. Significant difference from distilled water group

† -(*p*<0.05)

†† - (*p*<0.01); Significant difference from DMSO group

‡ - (*p*<0.05);

‡‡ - (*p*<0.01); Significant difference from MMS

* -(*p*<0.05)

** -(*p*<0.05); DDW-distilled water; MMS-methyl methane sulfonate (60 mg/kg); MNPCE-micronucleated polychromatic erythrocyte; PCE-polychromatic erythrocyte.

In the genotoxicity study, the % PCE in the MSM groups were significantly lower compared to the negative control group but not significantly different from the vehicle group, except at the 100 mg/kg concentration. In the MNL exposed groups, the % PCE was not significantly different from the vehicle groups but was significantly lower at 50 mg/kg and 100 mg/kg and significantly higher at 200 mg/kg and 400 mg/kg compared to the negative control. In the MLC exposed groups, % PCE was insignificantly different from both negative and vehicle control but was decreasing with increasing concentrations. In the antigenotoxicity group, % PCE in all the extracts+MMS treated groups was higher than in the MMS group.

### Sperm morphology assay

**Figure 2:**
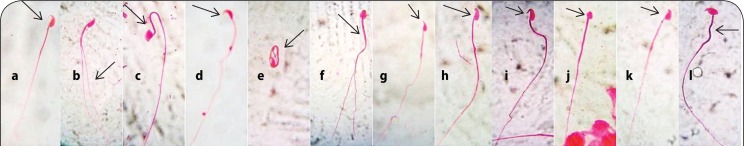
Abnormal sperm cells induced in mice exposed to different concentrations of aqueous and hydro-methanol extracts of *S. mombin, N.* *lotus* and *L. cylindrica* (a) normal sperm cell (b) sperm with two tails (c) amorphous head (d) banana head (e) folded (f) fused (g) hook at wrong angle (h) hookless (i) nubbed hook (j) pin head (k) short hook (l) wrong tail attachment. Magnification 1000x.

Compared to the negative control, a significant increase in abnormal sperm cells was observed at the concentrations of the aqueous extracts tested (except at the 1600 mg/kg of ASM; Table 3). In the ASM and ANL treated groups, there was a decrease in the frequency of aberrant sperm cells with increasing concentration, except at 400 mg/kg of ANL. The frequency of abnormalities was significantly lower at 1 600 mg/kg in ANL treated mice. In the hydro-methanol extract treated groups, the frequency of aberrant sperm cells was significantly lower than observed in the DMSO treated group in both MSM and MLC, except at 400 mg/kg of MLC (Table 3). However, in the MNL treated groups, there was a concentration dependent significant increase (except at the 50 mg/kg concentration) in frequency of aberrant sperm cells compared to the DMSO group. Figure 2 (a–l) shows the different types of abnormal sperm cells observed in the extract-treated mice.

**Table 3:** Frequency of abnormal sperm cells (Mean±Standard Error) induced in mice exposed to aqueous and hydro-methanol extracts of *Spondias mombin, Nymphaea lotus* and *Lufffa cylindrica*.

Treatment		Mean ± SE	Mean ± SE	Mean ± SE
Distilled water		31.8±1.3	31.8±1.3	31.81.3
DMSO		58.4±5.5^†^	41.0±10.9^†^	41.010.9^†^
MMS		83.8±26.7^†^	83.8±26.7^†^	83.826.7^†^
Aqueous extracts	Conc. (mg/kg)	ASM	ANL	ALC
	200	88.6±32.8^†^	85.3±40.8^†^	41.7±15.0^†^
	400	42.9±24.3^†^	92.6±36.5^†^	41.2±14.8^†^
	800	45.0±19.6^†^	66.9±28.7^†^	50.2±17.1^†^
	1600	31.3±23.7	27.4±26.2^†^	48.7±16.2^†^
Hydro-methanolic extracts	Conc. (mg/kg)	MSM	MNL	MLC
	50	26.2±2.0^†‡^	35.8±11.0^†‡^	23.8±6.7^†‡^
	100	39.6±1.9^†‡^	47.0±12.8^†‡^	33.6±8.8^‡^
	200	25.6±2.3^†‡^	48.0±19.5^†‡^	25.2±7.0^†‡^
	400	23.0±0.9^†‡^	76.8±33.5^†‡^	42.3±10.0^‡^

DMSO: dimethyl sulfoxide; MMS: methyl methane sulfonate, Conc: concentration; Significant difference from distilled water group

† -(*p*<0.05); Significant difference from DMSO group

‡ -(*p*<0.05)

## Discussion

Herbal medicines have become a popular form of therapy both in the developed and developing countries. They are believed to be non toxic, with little or no side effects compared to conventional drugs. Despite their widespread recognition, herbal medicines could cause toxicity due to their inherent properties, contamination, and variability of active or toxic components because of variations in culturing, processing, and preparation techniques (Tomlinson *et al*., 2000; Ko, 2004). Despite the common use of *S. mombin, N. lotus* and *L. cylindrica* in the Nigerian traditional management of diseases, information on the safety of these medicinal plants is limited. In the context of this fact, the current study was undertaken.

The acute toxicity study indicated that the extracts, each at a dose of 5 000 mg/kg p.o., did not cause mortality or any clinical sign of toxicity. We therefore concluded that the lethal dose (LD5_0_) of the tested extracts in mice, after a single oral administration is higher than 5 000 mg/kg under our experimental conditions. Loomis and Hayes (1996) and Rosidah *et al*. (2009) classified substances with LD5_0_ values 500–5 000 mg/kg as non-toxic and those with LD5_0_ values of 5 000–15 000 mg/kg as practically nontoxic. Thus these extracts can be considered non-toxic. This is in line with earlier reports that the aqueous extract of *S. mombin* up to 5 000 mg/kg p.o. (Ayoka *et al*., 2005; Hamenoo, 2010) and methanol extract of *L. cylindrica* up to 3 000 mg/kg (Hazra *et al*., 2011) are non-toxic.

*In vivo* micronucleus assay, a useful tool which has been widely used to evaluate genotoxicity, can detect both chromosomal damage and mitotic disturbances in peripheral blood cells or bone marrow cells. This assay has the advantage of circumventing the challenges of *in vitro* evaluation and providing more valuable information (Hwang *et al*., 2013). In the present study, we observed the ratio of PCE/NCE and the incidence of micronuclei formation in bone marrow cells. Our data showed a weak genotoxicity of ASM, while MSM was genotoxic in mouse somatic cells. This corroborates earlier studies where the aqueous extract of *S. mombin* was reported to induce weak genotoxicity in *Allium cepa* root tip cells (Oyeyemi & Bakare, 2013) and increased MNPCE in rats exposed to it for 30 consecutive days (Odunola *et al*., 2011). ANL, ALC and MLC were genotoxic while MNL was weakly genotoxic in mouse somatic cells. This is in line with the report of Sowemimo *et al*. (2007) who observed that hydro-ethanol extract of *N. lotus* induced chromosomal aberration in rat lymphocytes. The present findings imply that the extracts tested have the potential to induce somatic genotoxicity in the mouse and in other animals. The observed genotoxicity might be due to the presence of alkaloids and tannins in these extracts as preliminary phytochemical analysis of the extracts showed the presence of alkaloids, tannins, saponins, steroids, terpene, flavonoids, phenolics, anthraquinones and cardiac glycoside (Oyeyemi & Bakare, 2013). This may imply an interaction of one/more of the phytochemicals with DNA or with the mitotic apparatus. Several medicinal plants with many pharmacological activities have been reported to be genotoxic and/or mutagenic (Santos *et al*., 2012; Pillay *et al*., 2013; Alves *et al*., 2014; Ferreira-Machado *et al*., 2014). This is because crude extracts are composed of several phytochemicals which may work synergistically, antagonistically or additively.

MMS is a mono-functional alkylating agent, with the capacity to generate methylating and ethylating species that interact with macromolecules, such as DNA. It induced DNA double-strand break (Bakkali *et al*., 2005), micronucleus in mouse bone marrow (Leffa *et al*., 2012), and cancer in mice (Wahnschaffe *et al*., 2005). MMS is a known testicular toxicant; it induced abnormalities in mouse sperm head (Wyrobek & Bruce, 1975; Cassidy *et al*., 1983). The extracts tested were able to alleviate the genotoxic effect of MMS in mice to different degrees. The highest dose of ASM was able to completely reverse the genotoxic effect of MMS. Cells with genotoxic stress might have been killed so that the frequency of MNPCE was reduced in the group. This means that the extracts also possess some antigenotoxic potential. This is in line with the established report that many mutagenic or carcinogenic substances also show antimutagenic or anticarcinogenic potential (Zeiger, 2003). The antigenotoxic effect observed with these extracts may be due to prevention of the formation of active species of MMS, scavenging of the active species or free radicals produced by MMS, blocking of the binding sites of the active species or antioxidant effects of the extracts.

A decrease in % PCE was observed in both aqueous and hydro-methanol extracts. Krishna and Hayashi (2000) concluded that the PCE-to-NCE ratio between test agent-treated animals and vehicle control animals provides a cytotoxicity index. This is an indication that the phytochemical constituents of the extracts elevated the rate of aging of the erythrocytes from PCE to NCE, thereby decreasing their normal life span and increasing the risk of genotoxicity. However, the extracts were able to alleviate MMS induced cytotoxicity.

The sperm abnormality test is a sensitive and reliable endpoint to identify chemicals that induce spermatogenic dysfunction (Wyrobek & Bruce, 1980). The sperm head abnormalities may be the result of mistakes made in packaging the genetic material in the sperm head or perhaps the result of an abnormal chromosome complement (Wyrobek & Bruce, 1978). It is also a marker of other sperm defects that significantly impair fertility (Nikolettos *et al*., 1999). In this study, all the aqueous extracts induced significant increase in aberrant sperm cells. The hydro-methanol extracts of *S. mombin* and *L. cylindrica* significantly reduced the background frequency of aberrant sperm cells, while the hydro-methanol extract of *N. lotus* significantly increased it. This shows that the extract of *N. lotus* may be toxic to the germ line as both the aqueous and hydro-methanol extract induced increase in aberrant sperm cells in the mouse. The aqueous extract of *S. mombin* and *L. cylindrica* induced increase in sperm cell aberration which decreased with increasing concentrations, while the hydro-methanol extract significantly reduced the frequency of background aberrant sperm cell. This is in concert with Raji *et al*. (2006) who reported an increase in abnormal sperm cells of rats exposed to aqueous extract of *S. mombin* bark and Ola-Davies *et al*. (2014) who reported that the methanol fraction of *S. mombin* did not alter the sperm morphology of rats. The reduction of background aberrant sperm cells by these extracts might be due to their antioxidant properties (Du *et al*., 2006; Igwe *et al*., 2012, Rufino *et al*., 2011). The sperm abnormalities observed could be due to the induction of point mutations in the early spermatocytes and spermatogonia at the premeiotic stages of spermatogenesis (Hugenholtz & Bruce, 1983). Mutation in germ cells prior to or during the reproductive period can be transmitted to later generations resulting in reproductive defects (Taylor, 1980). This may lead to carcinogenicity or teratogenicity in somatic cells. It may also alter a gene so that it contains a wrong code (Aduloju *et al*., 2008). Moreover, oxidative damage has been implicated to be a major player in sperm damage (Aitken *et al*., 2009; Kothari *et al*., 2010; Zini & Al-Hathal, 2011). Reactive oxygen species (ROS) damage phosphatides of the cell membrane by peroxidized metabolites of fatty acids, thus damaging sperm function and morphology (Alvarez *et al*., 1987). The observed anomalies could also be due to the extracts producing pituitary-hypothalamic or sex hormonal effects, which in turn affect spermatogenesis or cause abnormalities in seminal fluid resulting in functional or structural impairment of sperms (Odeigah, 1997). This study showed that the extracts contained phytochemicals that were capable of interacting with the genetic processes involved in spermatogenesis in mice.

A difference was observed in the activities of the aqueous and hydro-methanol extracts both in MN and sperm morphology assays. This is due to the fact that different solvents have the capacity to extract different phytochemicals depending on the polarity or solubility of the phytochemical. This is in line with previous reports in studies where different solvents were used for extraction (Abozed *et al*., 2014; Idu *et al*., 2008; Mary & Begum, 2014).

Our study showed that the extracts, except the hydromethanol extracts of *S. mombin*, exhibited the potential to induce both somatic and germ line genetic damage, while they could also counteract the effect of known mutagens/carcinogens such as MMS in the experimental condition used. According to Ferguson (2001), there is evidence that certain compounds can both induce and prevent DNA damage. The beneficial and/or harmful effects of the natural medicinal products typically result from combinations of various phytochemicals present in the plant (Briskin, 2000; Ulrich-Merzenich *et al*., 2007). There is thus an urgent need to identify the substances responsible for the therapeutic effect and toxicity so as to enable orientation of a safe use of the plant (Ferreira-Machado *et al*., 2014).

We conclude that while these plants may have some therapeutic effect, they may also be harmful/toxic, especially when used for a long period of time. Hence caution should be applied in the use of these plants as herbal medicines.
